# Supplementary material for: Characterizing cancer metabolism from bulk and single-cell RNA-seq data using METAFlux
Source: Nat Commun. 2023 Aug 12;14:4883. doi: 10.1038/s41467-023-40457-w (PMC10423258; doi:10.1038/s41467-023-40457-w)
Supplement: Supplementary file 1 — Supplementary Information [file 41467_2023_40457_MOESM1_ESM.pdf]

Characterizing cancer metabolism from bulk and single-cell RNA-seq data using METAFlux

Yuefan Huang[1, 2], Vakul Mohanty[1], Merve Dede[1], Kyle Tsai[1], May Daher[3], Li Li[3], Katayoun Rezvani[3], Ken Chen [1*].

[1] Department of Bioinformatics and Computational Biology, The University of Texas MD Anderson Cancer Center, Houston, Texas, 77030, USA

[2] Department of Biostatistics & Data Science, School of Public Health, The University of Texas Health Science Center at Houston (UTHealth), Houston, Texas, 77030, USA

[3] Department of Stem Cell Transplantation and Cellular Therapy, The University of Texas MD Anderson Cancer Center, Houston, Texas, 77030, USA

[*] Corresponding author: Ken Chen, Email: kchen3@mdanderson.org

Supplementary methods

1. Cell line medium composition

We analyzed the nutrient profiles of Ham's F-12(Supplementary Data File S1). METAFlux input only considers the presence or absence of metabolites, disregarding their concentrations. Therefore, to ensure the validity of the 44 metabolites, we included only those present in our metabolic network model, essential components like water and oxygen, cofactors, and inorganic phosphate. Furthermore, we added fatty acids and vitamins typically found in serum to account for the nutrients provided by the serum supplementation commonly used in cell culture to support cell growth.

2. Metabolic flux data dimension reduction and clustering rationale

We performed cube root transformation on TCGA LUAD flux output to preserve the sign (direction) of fluxes and mitigate the data's skewness. The total number of reactions calculated for bulk RNA-seq data is 13,082. Many fluxes are not informative or correlated with other fluxes. Thus, for downstream analysis, we only selected informative fluxes. We used the VST (variance-stabilizing transformation) method to select 3000 variable features. After feature selection, we performed PCA on the selected feature. Next, we applied graph-based unsupervised clustering on PC-reduced space to organize samples into clusters. Finally, we used Uniform Approximation and Projection method (UMAP)[1], a non-linear dimension reduction technique, for data visualization.

3. Metabolic flux data dimension reduction and clustering coding procedures

Seurat package was utilized on TCGA metabolic fluxes to perform dimension reduction, clustering, and visualization[1]. To normalize fluxes data, we took the cubic root of fluxes to

preserve fluxes' sign (direction). Next, we skipped the NormalizeData() function and manually input cube root normalized fluxes in the normalized data slot. The top 3000 features from FindVariableFeatures() were used for further analysis. Then we scaled the data using ScaleData(). Subsequently, RunPCA(), FindNeighbors(),FindClusters(), RunUMAP() were performed. The UAMP visualizations were generated by DimPlot() function.

4. Dimension reduction on metabolic gene expression data

The rationale for gene expression data clustering is similar to the previously mentioned procedures. Seurat package was used on TCGA bulk RNA-seq to perform dimension reduction, clustering, and visualization[1]. We skipped the NormalizeData() function, and log(TPM+1) was manually put in the normalized slot in the Seurat object. Subsequently, we ran FindVariableFeatures(), ScaleData(), RunPCA(), FindNeighbors(),FindClusters(), RunUMAP() on bulk samples. The UAMP visualizations were generated by DimPlot() function.

5. Lung cancer bulk sorted data processing

We only selected LUAD and LUSC tumor types for METAFlux analysis for bulk-sorted data. For samples with multiple runs, the average gene expression profiles were calculated. Samples without full immune, endothelial, tumor, and fibroblast gene expression profiles were not included in METAFlux assessment.

6. Survival analysis

To evaluate the prognostic significance of the identified lung adenocarcinoma (LUAD) cancer clusters derived from either METAFlux or gene expression-based profiles, we performed a survival analysis using the TCGAanalyze_survival() function from the TCGAbiolinks package in R[2]. The overall survival refers to the time from diagnosis until death. Patients were stratified into

groups based on their cluster assignments, and overall survival was analyzed for each group. The log-rank test was utilized to compare survival distributions across the groups, and p-values were reported to assess the statistical significance of the differences in overall survival between the clusters.

7. Calculation of nutrient uptake percentage in TME

To calculate the percentage of specific nutrient uptake in a particular cell type, we divided the total uptake of that nutrient from each cell type by the total TME uptake of that nutrient. The total cell type uptake was calculated by using per cell average flux multiplied by cell type proportion. Total TME nutrient uptake was calculated by summing the total uptake of that nutrient from all cell types.

Supplementary Tables

Supplementary Table S1. The Cox regression model coefficients for TCGA LUAD samples. Reference group for HR calculation: Cluster [LUSC-like LUAD], ajcc pathologic n [N0], ajcc pathologic m [M0], ajcc pathologic t [T1], gender [female], prior treatment [No], race [american indian or alaska native].

Predictors	HR Estimates	CI	p[1]
Cluster [LUAD1]	0.64	0.44 – 0.93	**0.019**
ajcc pathologic n [N1]	2.07	1.44 – 2.97	**<0.001**
ajcc pathologic n [N2]	2.61	1.75 – 3.90	**<0.001**
ajcc pathologic n [N3]	0.00	0.00 – Inf	0.995
ajcc pathologic n [NX]	0.99	0.24 – 4.10	0.992
ajcc pathologic m [M1]	1.72	0.87 – 3.39	0.118
ajcc pathologic m [M1a]	0.00	0.00 – Inf	0.996
ajcc pathologic m [M1b]	4.44	1.18 – 16.69	**0.027**
ajcc pathologic m [MX]	0.96	0.66 – 1.41	0.855
ajcc pathologic t [T1a]	2.18	0.99 – 4.79	0.053
ajcc pathologic t [T1b]	1.28	0.58 – 2.85	0.542
ajcc pathologic t [T2]	1.83	1.13 – 2.96	**0.013**
ajcc pathologic t [T2a]	1.20	0.61 – 2.36	0.599
ajcc pathologic t [T2b]	1.29	0.51 – 3.25	0.595
ajcc pathologic t [T3]	3.15	1.70 – 5.83	**<0.001**
ajcc pathologic t [T4]	2.47	1.14 – 5.34	**0.022**
ajcc pathologic t [TX]	5.23	0.64 – 43.03	0.124
age at index	1.01	1.00 – 1.03	0.166
gender [male]	0.90	0.66 – 1.22	0.487
prior treatment [Not Reported]	0.00	0.00 – Inf	0.999
prior treatment [Yes]	11.65	3.44 – 39.41	**<0.001**
race [asian]	1182357.39	0.00 – Inf	0.998
race [black or african american]	4468570.50	0.00 – Inf	0.998
race [not reported]	6476596.71	0.00 – Inf	0.998
race [white]	5258119.94	0.00 – Inf	0.998
Observations	490		

[1] only p-values less than 0.05 will be highlighted in bold

Supplementary Table S2. The coefficients for linear regression model:

$$Hypoxia\_scores \sim Oxygen\_uptake + Glucose\_uptake + Lactate$$

	**hypoxia score winter**		
Predictors	Estimates	CI	p[2]
(Intercept)	-0.05	-0.12 – 0.02	0.162
Oxygen uptake	-0.19	-0.27 – -0.12	**<0.001**
Glucose uptake	0.32	0.25 – 0.40	**<0.001**
Lactate secretion	0.09	0.02 – 0.17	**0.014**
Observations	676		
R^2 / R^2 adjusted	0.137 / 0.133		

[2] only p-values less than 0.05 will be highlighted in bold

Supplementary Table S3. Metabolites identified in competing and cooperation mechanisms for patient lung cancer samples.

**Metabolites (Competing)**	**Metabolites(cooperating)**
Glucose, Isoleucine, Lysine, Methionine, Threonine, Tryptophan, O_2, Glutamine, Tyrosine, Proline, Aspartate, Fe^{3+}, Ornithine, Fructose, Folate, 2-hydroxybutyrate, Citrate, Carnitine, Hypoxanthine, Cystine	Histidine, Leucine, Phenylalanine, Valine, H_2O, Asparagine, Cysteine, Arginine, Serine, Glutamate, PO_4^{3-}, SO_4^{2-}, Na^+, K^+, Ca^{2+}, Glycerol, Acetate, Biotin, Galactose, Riboflavin, Pyridoxine, Cl^-, Malonate, Citrulline, Acetone, Creatine, Formate, Betaine, Myo-Inositol, Niacinamide, Pyridoxal, Succinate, Urea

Supplementary Table S4. Glycolysis pathway genes[3] from Reactome

NUP160, TPR, NDC1, PFKP, PKM, NUP133, PRKACA, ENO1, NUP37, GCKR, SEH1L, NUP50, AAAS, NUP188, RAE1, PGK1, NUP93, PPP2CB, GPI, PPP2R1A, GAPDHS, GCK, ENO3 NUP88, ALDOC, NUP98, NUP107, GAPDH, TPI1, ENO2, PPP2R5D, GNPDA1, NUP155, PPP2CA, PFKFB4,NUP43, PFKFB2, NUP153, NUP85, NUP214, NUP210, NUP42, ALDOB, PPP2R1B, NUP54, NUP58, PFKL, PRKACB, PKLR, ALDOA, PFKM, RANBP2, NUP205, HK1, SEC13, PFKFB1, ADPGK, HK2, HK3, NUP35, GNPDA2, PGAM2, PRKACG, PGM2L1, PFKFB3, PGK2, PGAM1, BPGM, PGP, POM121, NUP62, PKLR, POM121C, PRKACA

[3] HGNC gene names

Supplementary Figures

Supplementary Figure 1

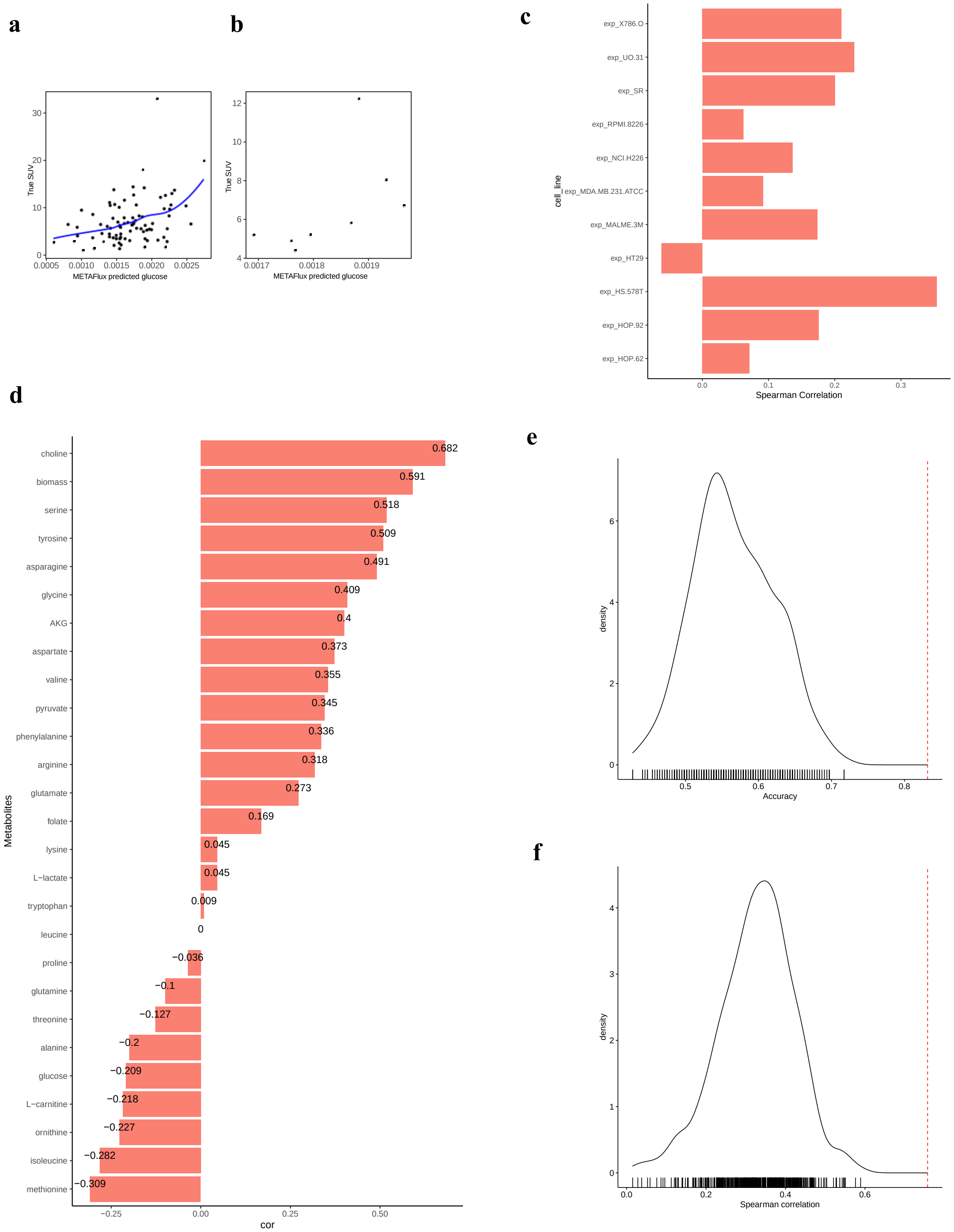

Supplementary Figure 1. SUV prediction and Simulation results of random medium and constraint-free medium predicted fluxes. **(a)** Scatter plot of Standardized Uptake Value (SUV) and METAFlux predicted glucose uptake for TNBC data (N=84). **(b)** Scatter plot of Standardized Uptake Value (SUV) and METAFlux predicted glucose uptake for pancreatic data (N=8). **(c)** Spearman correlation bar plot across each cell line for METAFlux model without any medium constraints. The spearman correlations between predicted fluxes and experimental fluxes were calculated for 11 cell lines. **(d)** Spearman correlation bar plot across each metabolite for METAFlux model without any medium constraints. The spearman correlations between predicted fluxes and experimental fluxes were calculated for 26 metabolites and one biomass reaction. **(e)** Distribution of correlations between predicted fluxes with experimental fluxes over 500 random mediums. The red dotted line indicates the results of the original biological meaningful medium. **(f)** Distribution of direction accuracies over 500 random mediums. The red dotted line indicates the result of the original biological meaningful medium.

Supplementary Figure 2

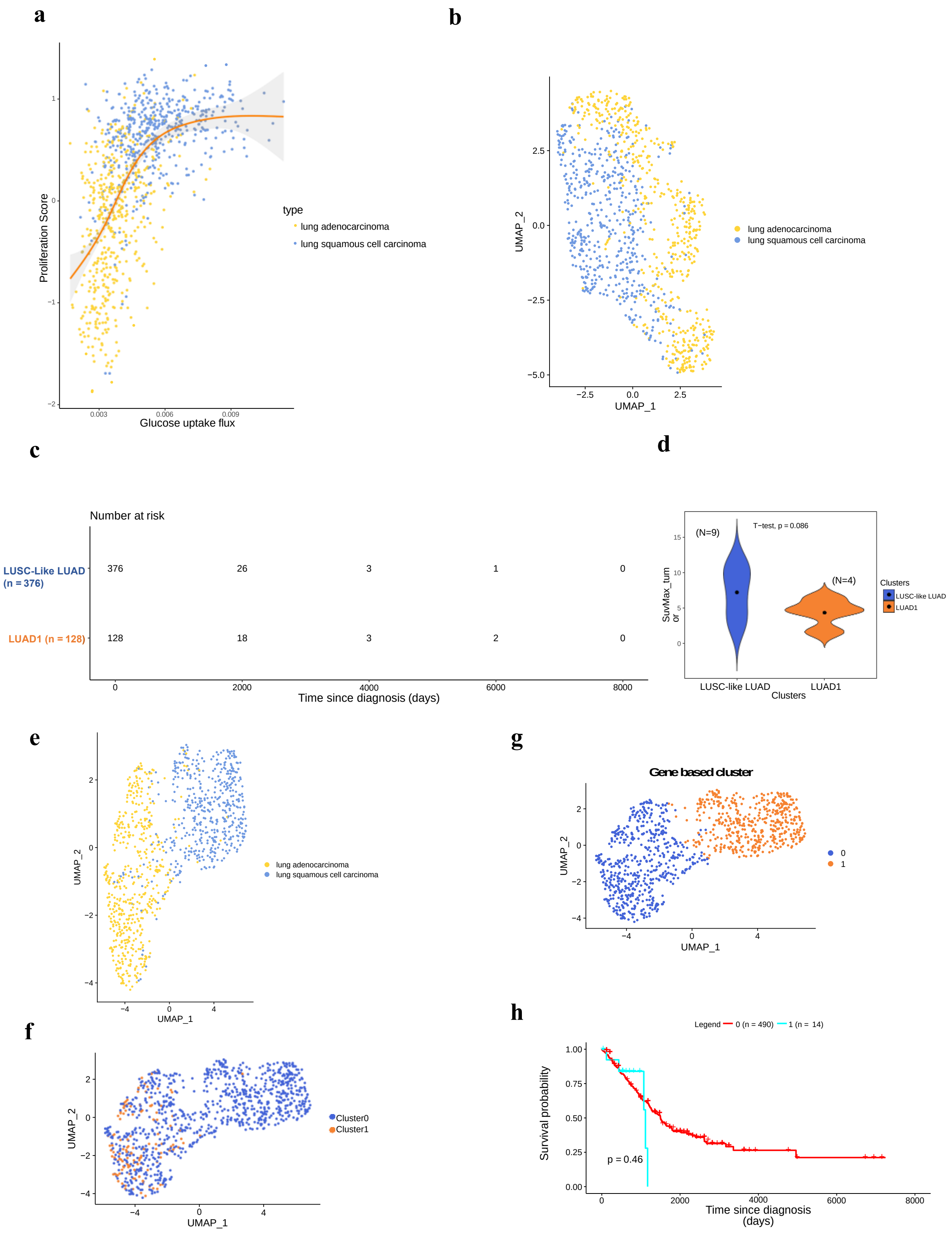

Supplementary Figure 2. Clustering and UMAP of TCGA LUAD and LUSC. (a) Scatter plot of glucose uptake flux with proliferation score. Each dot was color-coded by lung cancer type. The trend curve was generated using loess smoothing. **(b)** UMAP of TCGA LUAD and LUSC samples using predicted metabolic fluxes. Each dot represents one sample, and it is color-coded by lung cancer type. **(c)** Risk table for METAFlux predicted TCGA LUAD clusters. (**d**) SUV max values for the TCGA LUAD1 and LUSC-like LUAD patients. The two-sided p-value was calculated using T-test. (**e**) UMAP of TCGA LUAD and LUSC samples using metabolic gene expression. Each dot represents one sample, and it is color-coded by lung cancer type. **(f)** A gene-based clustering UMAP with flux-based clusters overlaid. **(g)** UMAP of TCGA LUAD and LUSC samples using metabolic gene expression data. **(h)** Overall Kaplan-Meier survival curves for clusters in TCGA LUAD. Clusters were generated using metabolic gene expression data.

Supplementary Figure 3

a

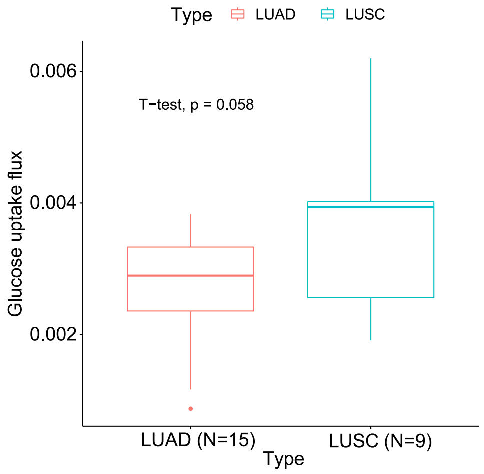

b

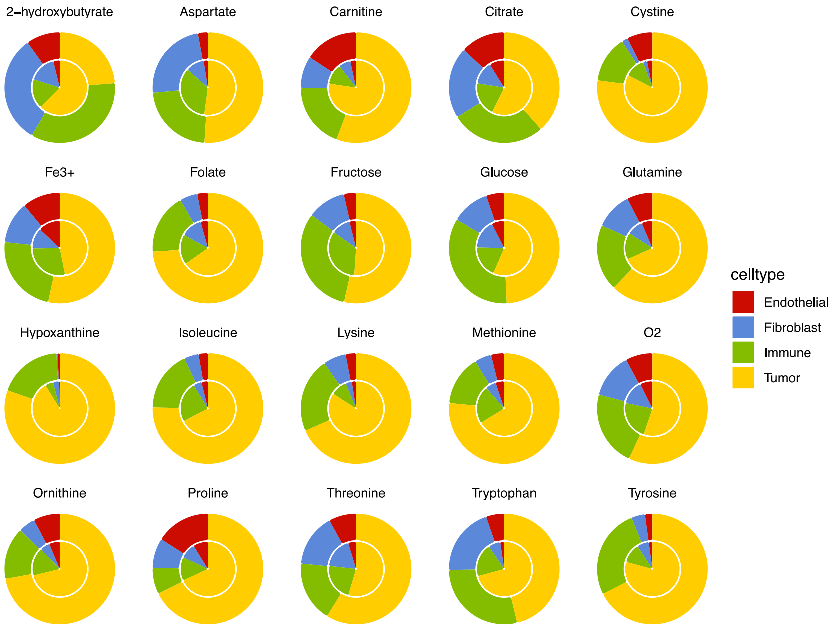

Supplementary Figure 3. METAFlux results on patient lung cancer datasets. (a) The overall TME glucose uptake comparison for LUAD (N=15 independent patient samples) and LUSC (N=9 independent patient samples). The box represents the interquartile range (IQR), line within shows the median, whiskers extend up to 1.5*IQR. Min and max values correspond to the smallest and largest mean predicted glucose flux. The two-sided p-value was calculated using T-test. **(b)** Donut pie charts of total cell type specific nutrient uptake percentage for LUAD and LUSC. The outer circle shows the LUSC nutrient uptake, and the inner circle shows LUAD nutrient uptake percentage. Cancer cells account for 56.55% of glucose uptake in LUAD and 49.28% of glucose uptake in LUSC. Immune cells account for 18.82% of glucose uptake in LUAD and 34.31% of glucose in LUSC. For glutamine, cancer cells account for 68.25% of glutamine uptake in LUAD and 62.32% of glutamine uptake in LUSC. Immune cells account for 15.79% of glutamine uptake in LUAD and 19.33 % of glutamine in LUSC.

Supplementary Figure 4

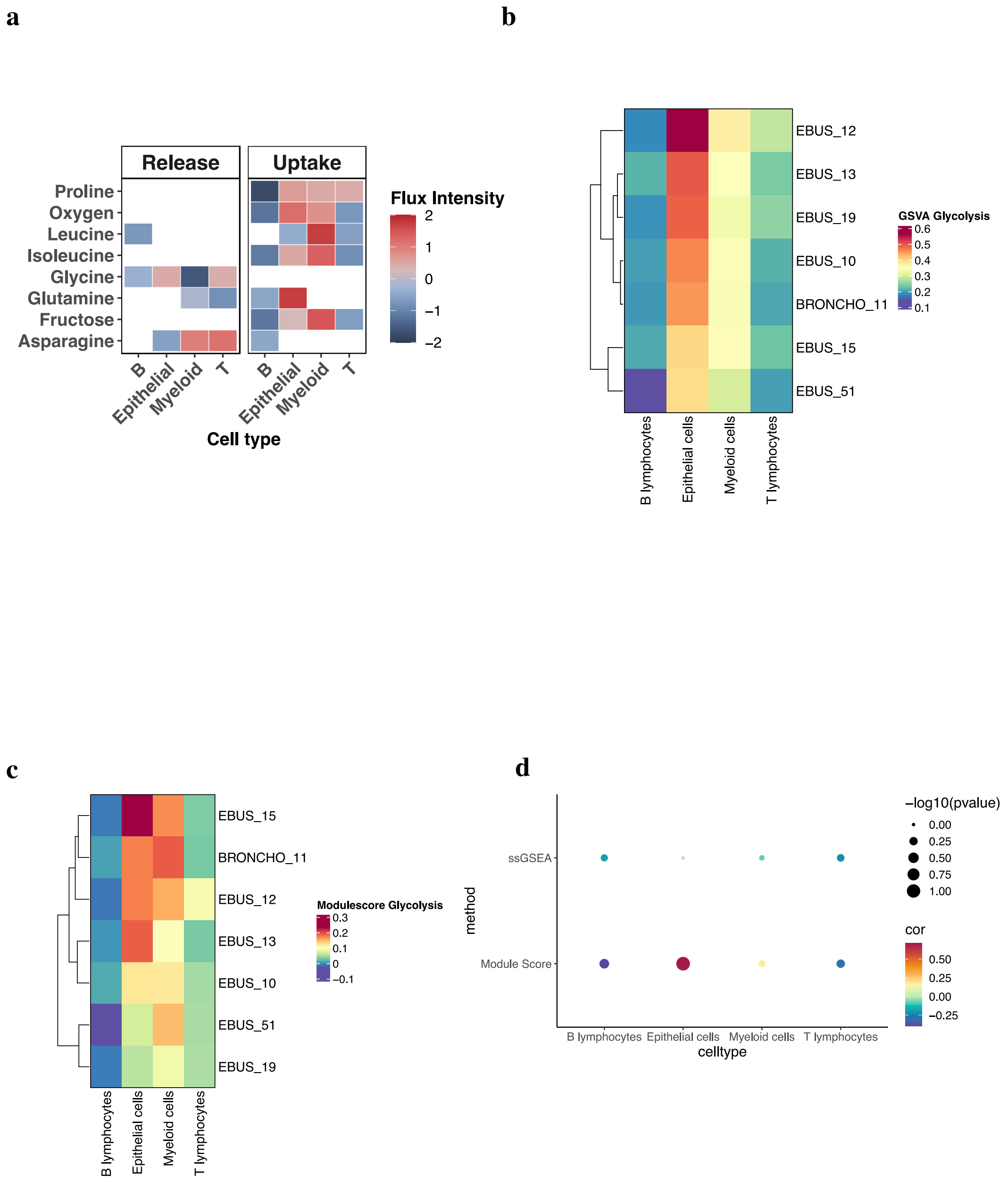

Supplementary Figure 4. METAFlux results on metastatic LUAD single-cell RNA-seq datasets. **(a)** The release and uptake profile of selected metabolites for each cell type. The color represents the absolute flux intensity. **(b)**Heatmap of ssGSEA (Single sample Gene Set Enrichment Analysis) glycolysis pathway scores of each cell type for seven patients. **(c)** Heatmap of Seurat AddModuleScore glycolysis pathway scores of each cell type for seven patients. **(d)** Dot plot showing the correlations between predicted glucose uptake flux and glycolysis pathway scores for a given cell type in seven patients. The color gradient of a dot represents correlation, and the size of a dot represents the P value.

Supplementary Figure 5

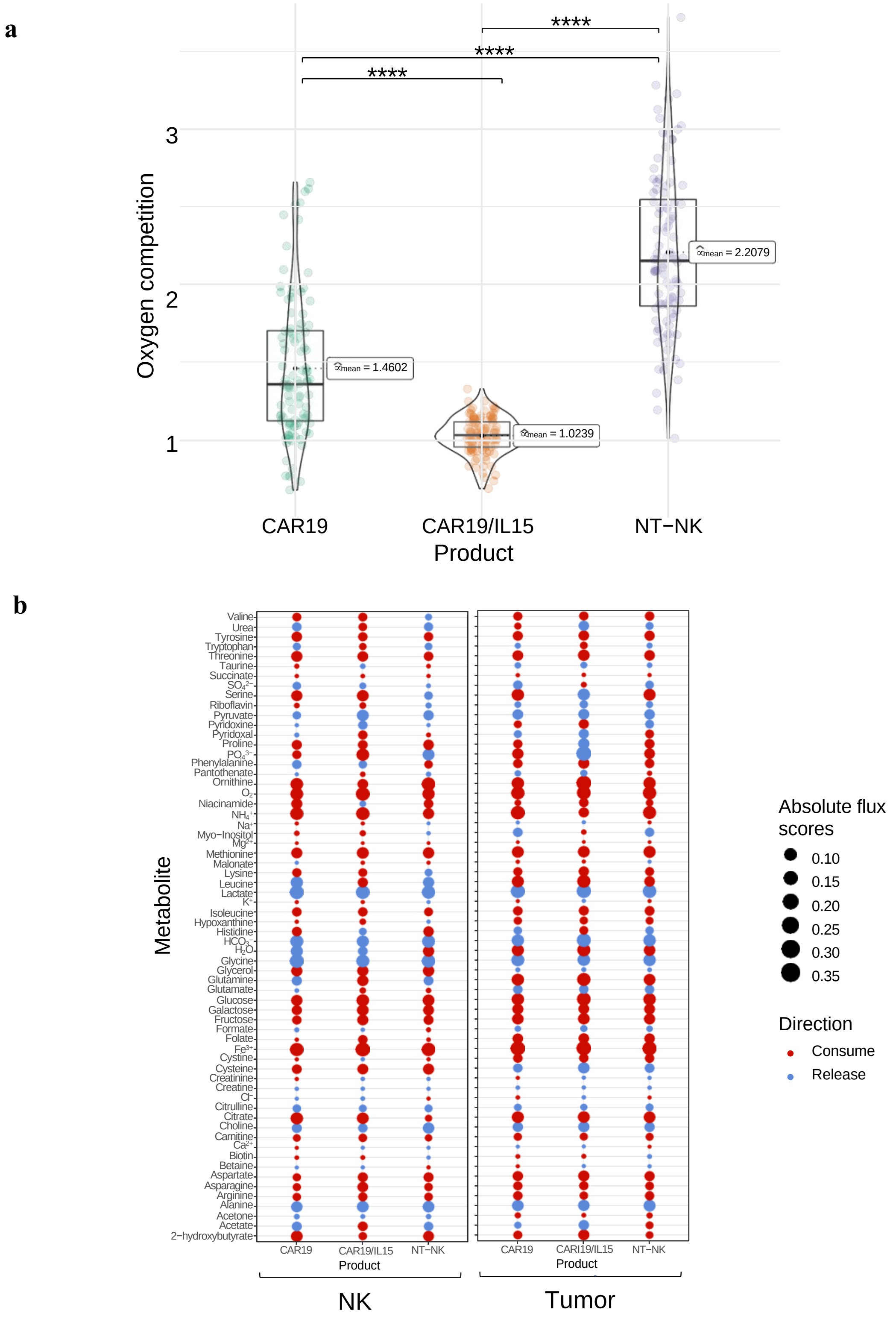

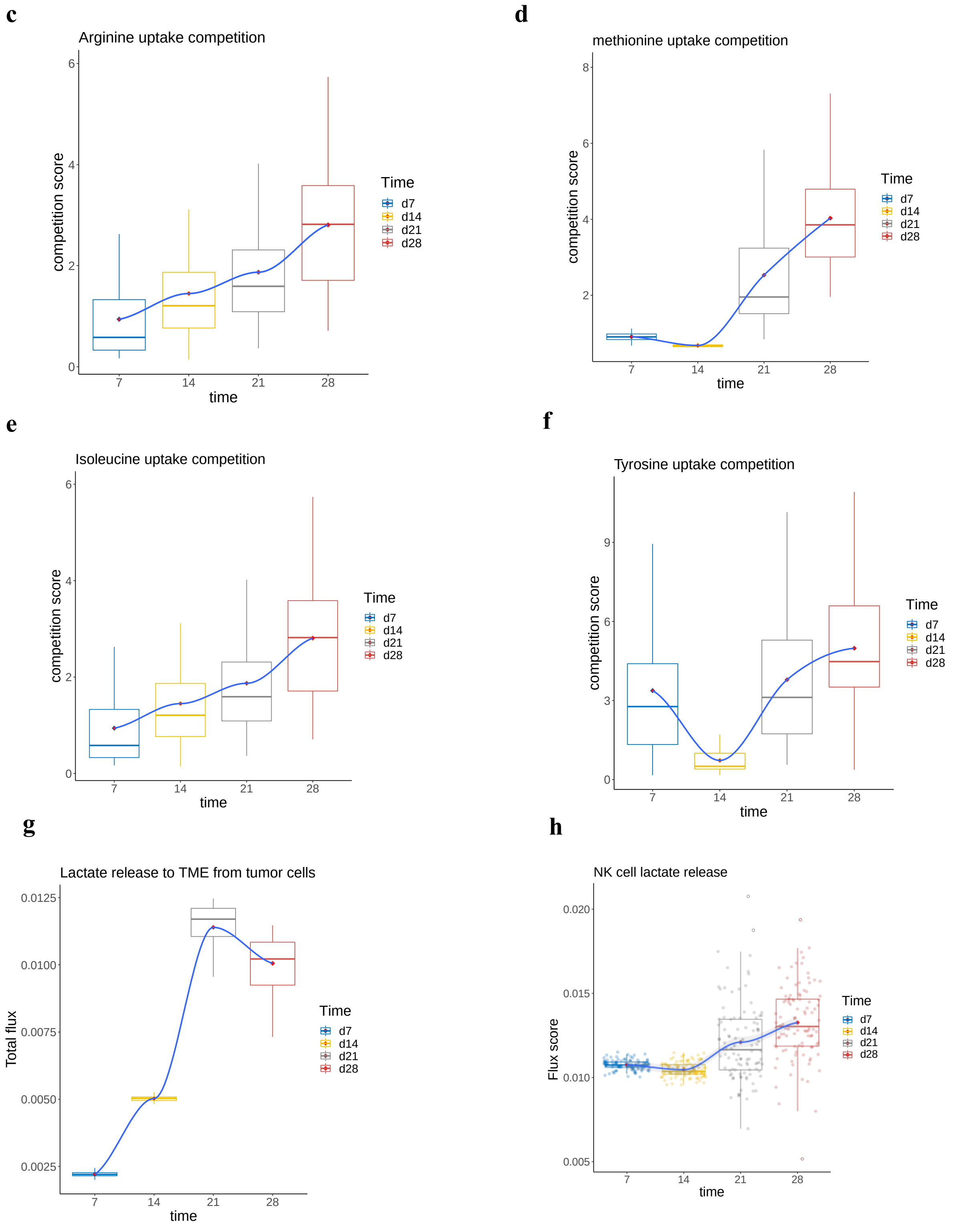
c
Arginine uptake competition
competition score
time
Time
d7
d14
d21
d28
d
methionine uptake competition
competition score
time
Time
d7
d14
d21
d28
e
Isoleucine uptake competition
competition score
time
Time
d7
d14
d21
d28
f
Tyrosine uptake competition
competition score
time
Time
d7
d14
d21
d28
g
Lactate release to TME from tumor cells
Total flux
time
Time
d7
d14
d21
d28
h
NK cell lactate release
Flux score
time
Time
d7
d14
d21
d28

Supplementary Figure 5. Nutrients profile and metabolic characteristics over time in CAR-NK datasets. (**a**) Competition scores of oxygen uptake fluxes for three products. The box within represents the interquartile range (IQR), the line within shows the median, and the whiskers extend up to 1.5*IQR. Each group includes n = 100 bootstrap samples. Min and max values correspond to the smallest and largest mean predicted flux after removing data points that fall more than three times the IQR beyond the quartiles (Q1 and Q3). Statistical test: two-sided Games Howell**.** P values are Holm corrected. P-values are denoted as follows: ns ($p > 0.05$), * ($p \leqslant 0.05$), ** ($p \leqslant 0.01$), *** ($p \leqslant 0.001$), **** ($p \leqslant 0.0001$). **(b)** NK cells and tumor cells nutrient profiles for each product. The nutrients shown were extracted using our defined human blood list. Dot size represents the mean absolute cubic root normalized flux scores, and color represents the direction of flux**. (c-h)** Boxplot of predicted flux over time. The box represents the interquartile range (IQR), the line within shows the median, and the whiskers extend up to 1.5*IQR. Each time point includes n = 100 bootstrap samples. Min and max values correspond to the smallest and largest mean predicted flux after removing data points that fall more than three times the IQR beyond the quartiles (Q1 and Q3). The grey band represents the 95% confidence interval for the loess (locally weighted scatterplot smoothing) curve. Competition score of **(c)** arginine, **(d)** methionine, **(e)** isoleucine, and **(f)** tyrosine for average cancer cell against average NK cell in TME from day 7 to day 28. **(g)** The trend plot of total tumor lactate release to TME from day 7 to day 28. **(h)** The trend plot of per cell lactate release by average NK cell over time.

Supplementary Note

1. ^{18}F-FDG PET-CT scans data from open-source TCIA (The Cancer Imaging Archive)

The study by Leitner, B. P. et al. utilized ^{18}F-FDG PET-CT scans data from open-source TCIA (The Cancer Imaging Archive) to examine glucose uptake in LUSC and LUAD tumors[3]. They found that the SUVmax normalized to SUV in the descending aorta was one-fold higher in LUSC than in LUAD. This was observed despite comparable body weight, stage, adipose tissue, or muscle volume. Notably, the researchers found that this effect was specific to the tumor rather than a systemic effect. No differences were observed in 18F-FDG uptake in non-tumor tissues, including the heart, liver, skeletal muscle (deltoid), adipose tissue (SubQ abdominal), and brain.

2. Subpopulation analysis of scRNA-seq metastatic LUAD tumors

We carried out additional subpopulation analysis on the single-cell lung cancer data[4] directly. We identified and analyzed metabolic characteristics for various immune cell subpopulations. For glucose, the subpopulation analysis revealed that all cell types exhibit an uptake of glucose, which suggests glucose competition exists in LUAD. The heterogeneous landscape of glucose uptake profiles highlights the complex interplay within the tumor microenvironment. Among all cell types, monocyte-derived macrophages (mo-Macs) and monocytes exhibited elevated glucose uptake, followed by MALT B cells (mucosa-associated lymphoid tissue B cells). In contrast, Follicular B cells and GC B cells in the DZ (germinal center (GC) B cells in the dark zones) showed lower glucose uptake. This heterogeneity in glucose uptake among B cell subsets reflected differences in their metabolic states within the LUAD microenvironment.

For oxygen, similarly, all cell types exhibited an uptake of oxygen, which suggests oxygen competition exists in LUAD. Mo-Mac cells showed the highest oxygen uptake, followed closely

by CD207+CD1a+ LCs and epithelial cells (tumor) cells. This suggests that these cell types rely more on oxidative phosphorylation for energy production. Moreover, data revealed a distinct release and uptake pattern of asparagine and isoleucine, underscoring the cooperative metabolic behavior among various cell types in LUAD. For example, most immune cell subsets, including cytotoxic CD8+ T, exhausted CD8+ T, CD8 low T, regulatory T (Treg), CD207+CD1a+ Langerhans cells (LCs), CD4+ T helper (Th), monocytes, MALT B cells, naive CD4+ T, and naive CD8+ T, primarily release asparagine. This release pattern implies that these immune cells may supply asparagine to the asparagine-consuming cells within the tumor microenvironment, thus fostering metabolic cooperation.

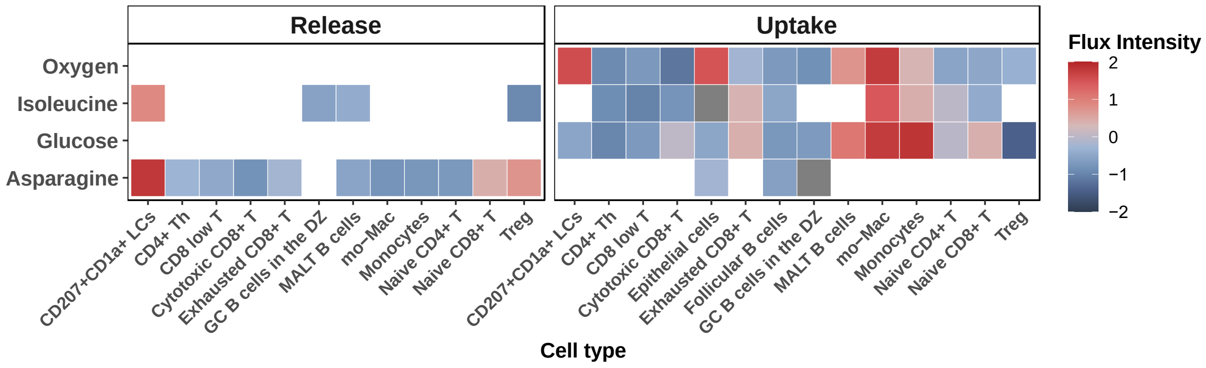

Supplementary Figure 6. The release and uptake profile of selected metabolites for each cell type. The color represents the absolute flux intensity. CD1c+ DCs (Langerhans cells, LCs), monocyte-derived macrophages (mo-Macs). GC B cells in the DZ (germinal center (GC) B cells in the dark zones which undergo rapid proliferation and somatic hypermutation), MALT B cells(MALT b cells mucosa-associated lymphoid tissue B cells).

3. Comparison between METAFlux with Compass

While both METAFlux and Compass employ a constrained-based approach to model single-cell data, they differ in their underlying assumption, objectives, resolutions, and use cases. We will highlight several main differences between each tool.

First, METAflux utilizes the Human1 metabolic mode to perform the FBA, while Compass employs Recon2. These different underlying metabolic models result in unique reaction IDs, reaction counts, and scopes.

Second, METAflux is specifically designed for analyzing metabolic profiles in the tumor context, capturing heterogeneity in the tumor microenvironment (TME) and elucidating metabolic interactions among different cell types by modeling all cell types as a single community. In contrast, Compass is a more general approach without constraints on the cellular community [5], because it computes maximal fluxes for metabolic reactions, assigns penalties based on mRNA expression, and determines flux distributions that minimize overall penalties while maintaining a specified flux threshold

Third, METAflux requires bootstrapping for single-cell data, resulting in cluster-wise metabolic statistics. It provides insights onto the collective metabolic behavior, focusing on the metabolic landscape and interactions among various cell types. In comparison, Compass directly outputs single-cell-wise fluxes. This difference in resolution also makes it difficult to perform direct comparison.

Lastly, METAflux emphasizes exchange flux profiles to understand metabolic interactions (cooperation, competition, etc.) among cell types in the TME for single-cell contexts, whereas Compass focuses more on non-exchange reaction fluxes. The downstream analysis illustrates different use cases, such as performing differential reaction activity analysis and computing

Cohen's d values, which can suggest the upregulation or downregulation of reactions between two distinct groups.

4. Metabolic enzyme expression validation

We have analyzed the expression of key metabolic genes from the GSE131907 lung cancer single-cell data and from GSE123902, an independent dataset[6]. The expression of these key metabolic genes is already encoded in the predicted fluxes derived from our algorithm. Previous research suggested that both *GLUT1* (*SLC2A1*) and *GLUT3* (*SLC2A3*) have positive correlations with FDG-PET SUV values in NSCLCs (non-small cell lung cancers)[7].

Thus, we extracted the expression of glucose transporter genes *SLC2A1* and *SLC2A3* from the original dataset and calculated the module scores for glucose uptake using the Seurat AddModuleScore function. The key gene signatures scores showed a consistent trend of glucose uptake predictions made by METAFlux, with the order being Myeloid cells > B cells > T cells > Epithelial cells. This consistency between gene expression profiles and our algorithm's predictions provides further support for the reliability of our model.

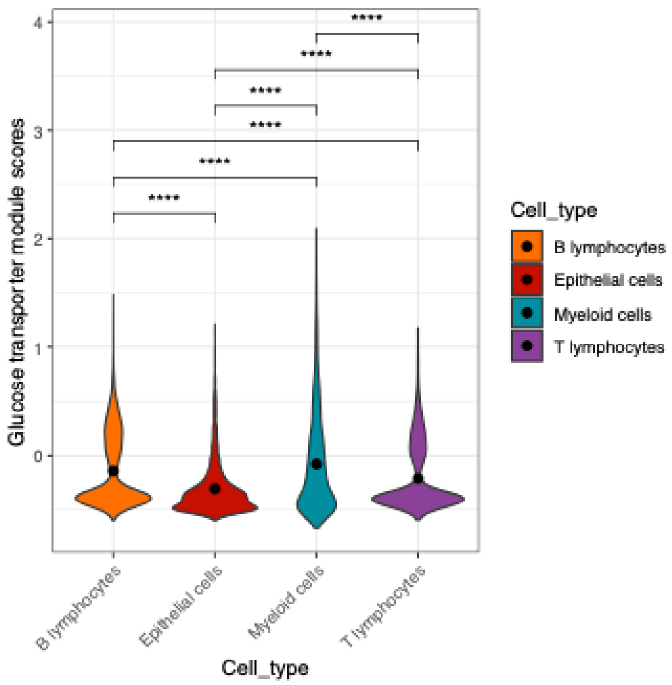

Supplementary Figure 7. Seurat AddModuleScore of glucose transporter genes *SLC2A1* and *SLC2A3* for four cell types in GSE131907 lung cancer single cell data.

The independent dataset (GSE123902) comprising brain (n = 3), bone (n = 1), and adrenal (n = 1) lung adenocarcinoma metastases[6], we extracted the expression of glucose transporter genes *SLC2A1* and *SLC2A3* and calculated the module scores.

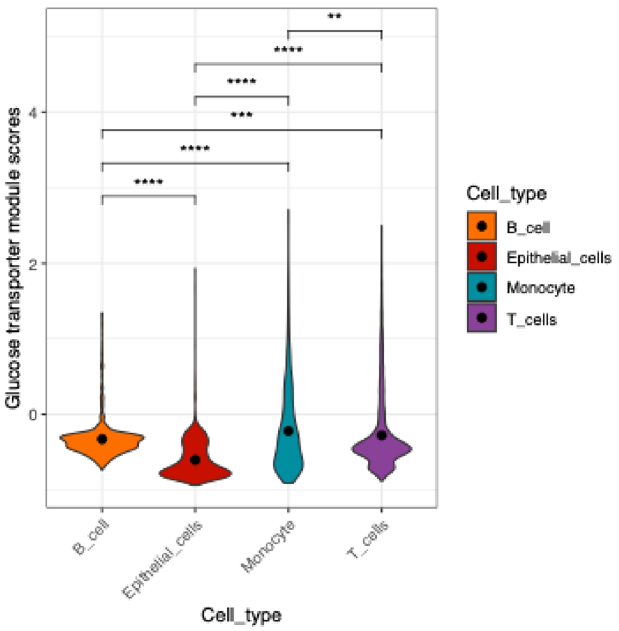

Supplementary Figure 8. Seurat AddModuleScore of glucose transporter genes *SLC2A1* and *SLC2A3* for four cell types in GSE123902 lung cancer single cell data.

Our analysis revealed a similar trend to our findings in metastatic lymph node samples, with myeloid cells exhibiting the highest glucose transporter scores and tumor cells having the lowest scores. The order between T cells and B cells is changed, which is likely attributable to sampling differences. Nevertheless, this observation consistently shows the highest glucose uptake in myeloid cells and the lowest glucose uptake in tumor cells, supporting the robustness of our finding.

References

1 Hao, Y. *et al.* Integrated analysis of multimodal single-cell data. *Cell* **184**, 3573-3587 e3529, doi:10.1016/j.cell.2021.04.048 (2021).

2 Colaprico, A. *et al.* TCGAbiolinks: an R/Bioconductor package for integrative analysis of TCGA data. *Nucleic Acids Res* **44**, e71, doi:10.1093/nar/gkv1507 (2016).

3 Leitner, B. P. *et al.* Multimodal analysis suggests differential immuno-metabolic crosstalk in lung squamous cell carcinoma and adenocarcinoma. *NPJ Precis Oncol* **6**, 8, doi:10.1038/s41698-021-00248-2 (2022).

4 Kim, N. *et al.* Single-cell RNA sequencing demonstrates the molecular and cellular reprogramming of metastatic lung adenocarcinoma. *Nat Commun* **11**, 2285, doi:10.1038/s41467-020-16164-1 (2020).

5 Wagner, A. *et al.* Metabolic modeling of single Th17 cells reveals regulators of autoimmunity. *Cell* **184**, 4168-4185 e4121, doi:10.1016/j.cell.2021.05.045 (2021).

6 Laughney, A. M. *et al.* Regenerative lineages and immune-mediated pruning in lung cancer metastasis. *Nat Med* **26**, 259-269, doi:10.1038/s41591-019-0750-6 (2020).

7 Meyer, H. J., Wienke, A. & Surov, A. Associations between GLUT expression and SUV values derived from FDG-PET in different tumors-A systematic review and meta analysis. *PLoS One* **14**, e0217781, doi:10.1371/journal.pone.0217781 (2019).
