# Supplementary material for: Characterizing cancer metabolism from bulk and single-cell RNA-seq data using METAFlux
Source: Nat Commun. 2023 Aug 12;14:4883. doi: 10.1038/s41467-023-40457-w (PMC10423258; doi:10.1038/s41467-023-40457-w)
Supplement: Supplementary file 3 — Description of Additional Supplementary Files [file 41467_2023_40457_MOESM3_ESM.pdf]

Description of Additional Supplementary Files

Supplementary Data File S1
Description: Cell line and human blood medium metabolite profile.

Supplementary Data File S2
Description: TNBC and pancreatic cancer glucose uptake SUV data and METAFlux glucose uptake prediction.
