# Supplementary material for: Characterizing cancer metabolism from bulk and single-cell RNA-seq data using METAFlux
Source: Nat Commun. 2023 Aug 12;14:4883. doi: 10.1038/s41467-023-40457-w (PMC10423258; doi:10.1038/s41467-023-40457-w)
Supplement: Supplementary file 6 — Reporting Summary [file 41467_2023_40457_MOESM6_ESM.pdf]

nature portfolio

Corresponding author(s): Ken Chen

Last updated by author(s): 07/05/2023

Reporting Summary

Nature Portfolio wishes to improve the reproducibility of the work that we publish. This form provides structure for consistency and transparency in reporting. For further information on Nature Portfolio policies, see our Editorial Policies and the Editorial Policy Checklist.

Statistics

For all statistical analyses, confirm that the following items are present in the figure legend, table legend, main text, or Methods section.

n/a	Confirmed	
☐	☒	The exact sample size (*n*) for each experimental group/condition, given as a discrete number and unit of measurement
☐	☒	A statement on whether measurements were taken from distinct samples or whether the same sample was measured repeatedly
☐	☒	The statistical test(s) used AND whether they are one- or two-sided<br>*Only common tests should be described solely by name; describe more complex techniques in the Methods section.*
☒	☐	A description of all covariates tested
☐	☒	A description of any assumptions or corrections, such as tests of normality and adjustment for multiple comparisons
☐	☒	A full description of the statistical parameters including central tendency (e.g. means) or other basic estimates (e.g. regression coefficient) AND variation (e.g. standard deviation) or associated estimates of uncertainty (e.g. confidence intervals)
☐	☒	For null hypothesis testing, the test statistic (e.g. *F*, *t*, *r*) with confidence intervals, effect sizes, degrees of freedom and *P* value noted<br>*Give P values as exact values whenever suitable.*
☒	☐	For Bayesian analysis, information on the choice of priors and Markov chain Monte Carlo settings
☒	☐	For hierarchical and complex designs, identification of the appropriate level for tests and full reporting of outcomes
☐	☒	Estimates of effect sizes (e.g. Cohen's *d*, Pearson's *r*), indicating how they were calculated

Our web collection on statistics for biologists contains articles on many of the points above.

Software and code

Policy information about availability of computer code

Data collection	TCGAbiolinks (Version '2.18.0') was used to obtain the TCGA clinical data. All other datasets are publicly available
Data analysis	TCGAanalyze_survival function in TCGAbiolinks(Version '2.18.0') was used to conduct survival analysis. The function ggbetweenstats from ggstatsplot (Version '0.7.0') was used to conduct graph and statistical analysis between groups. Seurat V4 was used to generate single-cell UMAP and gene expression plot. The remaining calculation was performed using METAFlux (https://github.com/KChen-lab/METAFlux). Other stofware packages were used to facilitate the plotting, analysis and data cleanning: RColorBrewer_1.1-2, pheatmap_1.0.12, readr_1.4.0 , gridExtra_2.3 , SummarizedExperiment_1.20.0, Biobase_2.50.0 ,ggpubr_0.4.0 , scales_1.1.1, ggplot2_3.3.3 ,biomaRt_2.46.3 , stringr_1.4.0 stringi_1.7.8 , tidyr_1.1.2,data.table_1.14.0, SingleCellExperiment_1.12.0, rstatix_0.7.0,reshape2_1.4.4.

For manuscripts utilizing custom algorithms or software that are central to the research but not yet described in published literature, software must be made available to editors and reviewers. We strongly encourage code deposition in a community repository (e.g. GitHub). See the Nature Portfolio guidelines for submitting code & software for further information.

Data

Policy information about availability of data

All manuscripts must include a data availability statement. This statement should provide the following information, where applicable:

- Accession codes, unique identifiers, or web links for publicly available datasets
- A description of any restrictions on data availability
- For clinical datasets or third party data, please ensure that the statement adheres to our policy

All datasets used in our study are publicly available. Human-GEM model was accessed from (https://github.com/SysBioChalmers/Human-GEM). We retrieved the medium composition and experimental flux profiling data for 11 NCI-60 cell lines under the original manuscript 53. ecGEM flux prediction for 11 NCI-60 cell lines was be obtained at https://zenodo.org/record/3583004#.YhQJdZPMJqs. NCI-60 cell lines TPM RNA-seq data was obtained from https://depmap.org/portal/download/. TNBC FDG-PET data and matched expression data can be found at NCBI Gene Expression Omnibus (GSE135565) [https://www.ncbi.nlm.nih.gov/geo/query/acc.cgi?acc=GSE135565], and pancreatic cancer FDG-PET data and matched expression can be downloaded at NCBI Gene Expression Omnibus (GSE107754) [https://www.ncbi.nlm.nih.gov/geo/query/acc.cgi?acc=GSE107754]. The TCGA pan-cancer RNA-seq TPM data was downloaded from UCSC Xena data hubs(https://xenabrowser.net/). The proliferation score data can be found in the original publication64. Patient Lung cancer bulk-sorted RNA-seq data can be downloaded from the NCBI Gene Expression Omnibus (GSE111907) [https://www.ncbi.nlm.nih.gov/geo/query/acc.cgi?acc=GSE111907]. Single-cell Metastatic LUAD data was retrieved from NCBI Gene Expression Omnibus (GSE131907) [https://www.ncbi.nlm.nih.gov/geo/query/acc.cgi?acc=GSE131907]. CAR-NK single-cell RNA-seq data is available through NCBI Gene Expression Omnibus (GSE190976) [https://www.ncbi.nlm.nih.gov/geo/query/acc.cgi?acc=GSE190976].

Human research participants

Policy information about studies involving human research participants and Sex and Gender in Research.

Reporting on sex and gender	Reporting sex and gender is not relevant in our study
Population characteristics	Population characteristics is not relevant to our study
Recruitment	Recruitment of human participants is not relevant to our study
Ethics oversight	Ethics oversight is not relevant to our study.

Note that full information on the approval of the study protocol must also be provided in the manuscript.

Field-specific reporting

Please select the one below that is the best fit for your research. If you are not sure, read the appropriate sections before making your selection.

[x] Life sciences [ ] Behavioural & social sciences [ ] Ecological, evolutionary & environmental sciences

For a reference copy of the document with all sections, see nature.com/documents/nr-reporting-summary-flat.pdf

Life sciences study design

All studies must disclose on these points even when the disclosure is negative.

Sample size	No prior sample size calculation was performed, because all datasets used in this manuscript are publicly available. The choice of datasets was driven by the quality of datasets and main story line of our manuscript.
Data exclusions	For NCI-60 cell lines, we only select 11 high quality cell lines and exclude the remaining cell lines due to possible nutreint depletion. For data GSE111907, We only selected LUAD and LUSC tumor types for METAFlux analysis. For samples with multiple runs, the average gene expression profiles were calculated. Samples without full immune, endothelial, tumor, and fibroblast gene expression profiles were not included in METAFlux assessment. For GSE131907, we removed cell type if population is less than 5% and removed low quality cells. For GSE190976, we removed possible doublets and low quality cells based on the nFeature, nCount and MT%.
Replication	We confirmed we could replicate findings under the same data quality metrics and random seeds.
Randomization	Allocation was not random.
Blinding	All results are based on public datasets, thus blinding was not performed.

Reporting for specific materials, systems and methods

We require information from authors about some types of materials, experimental systems and methods used in many studies. Here, indicate whether each material, system or method listed is relevant to your study. If you are not sure if a list item applies to your research, read the appropriate section before selecting a response.

Materials & experimental systems

n/a	Involved in the study
☒	☐ Antibodies
☒	☐ Eukaryotic cell lines
☒	☐ Palaeontology and archaeology
☒	☐ Animals and other organisms
☒	☐ Clinical data
☒	☐ Dual use research of concern

Methods

n/a	Involved in the study
☒	☐ ChIP-seq
☒	☐ Flow cytometry
☒	☐ MRI-based neuroimaging
